# How do sphingosine-1-phosphate affect immune cells to resolve inflammation?

**DOI:** 10.3389/fimmu.2024.1362459

**Published:** 2024-02-28

**Authors:** Gehui Sun, Bin Wang, Xiaoyu Wu, Jiangfeng Cheng, Junming Ye, Chunli Wang, Hongquan Zhu, Xiaofeng Liu

**Affiliations:** ^1^ The First Clinical College, Gannan Medical University, Ganzhou, Jiangxi, China; ^2^ Department of Critical Care Medicine, The First Affiliated Hospital of Gannan Medical University, Ganzhou, Jiangxi, China; ^3^ Clinical College, Suzhou Medical College of Soochow University, Suzhou, Jiangsu, China; ^4^ Department of Emergency, The First Affiliated Hospital of Gannan Medical University, Ganzhou, Jiangxi, China

**Keywords:** S1P, immune cells, SphKs, inflammation, signal pathway

## Abstract

Inflammation is an important immune response of the body. It is a physiological process of self-repair and defense against pathogens taken up by biological tissues when stimulated by damage factors such as trauma and infection. Inflammation is the main cause of high morbidity and mortality in most diseases and is the physiological basis of the disease. Targeted therapeutic strategies can achieve efficient toxicity clearance at the inflammatory site, reduce complications, and reduce mortality. Sphingosine-1-phosphate (S1P), a lipid signaling molecule, is involved in immune cell transport by binding to S1P receptors (S1PRs). It plays a key role in innate and adaptive immune responses and is closely related to inflammation. In homeostasis, lymphocytes follow an S1P concentration gradient from the tissues into circulation. One widely accepted mechanism is that during the inflammatory immune response, the S1P gradient is altered, and lymphocytes are blocked from entering the circulation and are, therefore, unable to reach the inflammatory site. However, the full mechanism of its involvement in inflammation is not fully understood. This review focuses on bacterial and viral infections, autoimmune diseases, and immunological aspects of the Sphks/S1P/S1PRs signaling pathway, highlighting their role in promoting intradial-adaptive immune interactions. How S1P signaling is regulated in inflammation and how S1P shapes immune responses through immune cells are explained in detail. We teased apart the immune cell composition of S1P signaling and the critical role of S1P pathway modulators in the host inflammatory immune system. By understanding the role of S1P in the pathogenesis of inflammatory diseases, we linked the genomic studies of S1P-targeted drugs in inflammatory diseases to provide a basis for targeted drug development.

## 1 Introduction

Inflammation is the body’s direct response to tissue and cell damage by pathogens, harmful stimuli (such as chemicals), or physical damage (1). As a defense mechanism, inflammation can quickly resist the invasion of foreign pathogens through an acute inflammatory response, and the organs and tissues of the body can be repaired. However, excessive inflammatory stimulation will cause imbalance of immune response (2). With an in-depth exploration of the mechanism of diseases, researchers have gradually realized that inflammation is linked to almost all human diseases and is crucial for maintaining homeostasis (3). Immune cells play important roles in inflammation occurrence and resolution. On the one hand, inflammation locally recruits many immune cells (such as neutrophils, macrophages, etc.) (4, 5), and immune cells are activated to release inflammatory factors (such as TNF-α, IL-6, IL-1β, etc.), leading to inflammation aggravation (6, 7). On the other hand, the resolution of the inflammatory response requires immune cells to release anti-inflammatory factors (such as IL-10, etc.) (8, 9). With the use of inflammatory cytokine blockers, the rapid progress of immune-targeted therapy drugs has been promoted. The targeted scope includes cytokines and their receptors, inflammatory cell transport, cell regulatory ligand receptors, and cell depletion strategies (10–12), which provide new directions for inflammation treatment.

Endogenous bioactive lipids are generally involved in the biological processes of human health and diseases and have been extensively studied in inflammation. They are involved in cell membrane formation and development, immune cell trafficking, and inflammatory cascade reactions (13). Sphingosine-1-phosphate (S1P) is a biologically active lysophosphatide that exerts a wide range of biological effects by binding to five different G protein-coupled receptors (GPCRs), regulating cell survival and migration, immune cell recruitment, angiogenesis, and lymphangiogenesis (14). S1P formation and degradation are tightly regulated in cells and are activated by sphingosine kinases (SphKs), followed by dephosphorylation by phosphatases (SPPs) or degradation by S1P lyases (SPL) (15, 16). Clinical studies have found that S1P levels are closely correlated with the severity of infection (17), and S1P plays various roles in immune responses. Therefore, it is important to understand how S1P participates in inflammatory responses (18). In this review, we focus on the effects of S1P on innate and adaptive immunity, discuss its effects on inflammation, and highlight recommendations to guide this area of research.

## 2 S1P signaling in inflammation

With the development of metabolomics, researchers have gradually realized that inflammation originates from various diseases, such as autoimmune diseases, cancer, sepsis, and diabetes, by exploring disease physiology and have found that inflammation increases susceptibility to diseases (19, 20). Recently, an in-depth exploration of the anti-inflammatory effect of sphingolipids has revealed that S1P in sphingolipids is closely associated with inflammatory signal transduction, such as TNF-α and TLRs (21). In S1P knockout mice, endothelial cell destruction and mortality were significantly increased after inflammatory stimulation, which could be reversed by restoring S1P levels by plasma infusion, indicating that S1P limited vascular leakage to maintain normal vascular integrity. SphKs are also required to prevent the lethal response of inflammatory mediators induced by vascular leakage (22). In a study on sepsis and systemic inflammatory response syndrome (SIRS), the plasma concentration of apolipoprotein M (apoM) was decreased in patients with sepsis and SIRS than in controls. The degree of the decrease was related to the severity of sepsis. It is speculated that a decrease in apoM may aggravate vascular leakage during infection (23). S1P has been shown to be a potential serum sphingolipid biomarker in sepsis, neuroinflammation, uterine inflammation, pneumonia, Wilson’s disease, hemolysis, cardiac and renal insufficiency, angiogenesis, etc. (24–26), and S1P is closely related to inflammatory markers, and generally S1P reduces the level of inflammatory markers. It has shown its role in endothelial protection and inflammation reduction (27, 28). In a single-center multi-case observational study, the serum S1P level of the healthy control group was (1156 ± 17 nmol/l), and the average serum S1P level of sepsis patients (580 ± 24 nmol/l) was significantly lower than that of the control group (29). This result is consistent with the results of other researchers (30, 31). S1P levels are closely related to a variety of diseases, current studies have shown that plasma S1P levels are decreased in various inflammation-related diseases (such as atherosclerosis, viral infection, sepsis), but in other diseases, such as type 2 diabetes mellitus, obesity, acute ischemic stroke, Alzheimer’s disease, multiple sclerosis, angina pectoris, heart failure, etc. Plasma S1P levels increased (31–33).

Sphingosine kinases type 1 and type 2 (Sphk1/2) are required to produce the immunomodulator S1P. Initially, researchers examined the inflammatory response in Sphk1 and Sphk2 gene knockout mice and were unable to elucidate the inflammatory effect of S1P; therefore, it was unclear whether S1P was pro-inflammatory or anti-inflammatory (14). With the continuous development of chemical and genetic tools, more mature knockout or overexpression mouse models of genes (Sphks and S1PRs) regulating S1P levels have been constructed, and regulating S1P and S1PRs biology has gradually progressed to clinical treatment of diseases (34, 35). In clinical observational studies, S1P levels were found to be significantly reduced in the serum of patients with sepsis and were inversely correlated with disease severity. Because S1P is a potent regulator of endothelial integrity, low S1P levels may lead to capillary leakage, impaired tissue perfusion, and organ failure in sepsis (29). During sepsis, inhibiting Sphks and S1P production can restore the secretion of thymic T cells and may improve sepsis prognosis (36). Subsequent studies have depicted that plasma S1P levels can predict mortality in sepsis and that platelet Sphk activity is positively correlated with plasma S1P concentrations in patients with sepsis (37). During inflammation, infiltrating immune cells and pro-inflammatory cytokine production increase endothelial permeability, and S1P signaling strengthens endothelial adhesion junctions to limit the progressively amplified inflammatory response (38). By regulating the expression of Sphk1 and S1P, kaempferol can inhibit the NF-kb pathway, reduce the expression of the inflammatory mediators NO and PGE2, alleviate the inflammatory response of LPS-induced sepsis, and stabilize the pulmonary vascular endothelial barrier (39). As the role of S1P in sepsis has been gradually widely studied, it has been proved that HDL-S1P in patients with sepsis has protective endothelial function and therapeutic potential through *in vivo* and *in vitro* experiments and observation (40). Among them, high-density lipoprotein (HDL) is the main carrier of S1P in plasma, while apoM binds to HDL through its retained signal peptide and is the carrier of HDL-S1P. In plasma, 60% of S1P is normally bound to apoM and the remaining 40% to albumin (41).

## 3 Role of Sphk1 and Sphk2 in inflammation

While exploring the role of S1P, we found that S1P exerts pro-inflammatory or anti-inflammatory effects depending on its upstream and downstream pathways. For example, Sphk1 and Sphk2 have completely opposite effects on sphingolipid metabolism (42). Sphk1 is localized in the cytoplasm and is transferred to the plasma membrane or secreted into the extracellular matrix after activation. However, Sphk2 is expressed in the endoplasmic reticulum, mitochondria, and nucleus (43). Sphk1 is a cytoplasmic enzyme that translocates to the cell membrane in response to various cellular stimuli, including immune attacks. This translocation is thought to promote S1P export into the extracellular space, where it binds and activates S1P receptors (S1PRs) in an autocrine or paracrine manner (44). Sphk1 is a pro-inflammatory factor, and inhibition of Sphk1 can inhibit the activation of NLRP3 inflammasome and the release of IL-1β in macrophages and improve the survival rate and pulmonary vascular leakage of cecal ligation and puncture (CLP)-induced sepsis mice (45). Moreover, Sphk1^–/–^ mice alleviated acetaminophen-induced endoplasmic reticulum (ER) stress and mitochondrial permeability changes and significantly reduced liver injury and inflammatory responses. In addition, Sphk1 deficiency reduced the level of histone deacetylase and promoted the acetylation of p65 and STAT1, thereby weakening the transcription of inflammatory genes (46). Sphk1-s1p signaling can activate the classical inflammatory pathway of NF-κB through the pro-inflammatory cytokine TNF-α (47). For example, recent studies have found that baicalin inhibits inflammation, oxidative stress, and apoptosis by inhibiting the Sphk1/S1P/NF-κB signaling pathway (48). Sphk1 is also a therapeutic target for various autoimmune diseases and gastrointestinal cancer (49, 50).

However, less research has been conducted on Sphk2 than Sphk1. The severity of colitis is increased by elevated COX-2 levels in Sphk2^–/–^ mice, and Sphk2 depletion enhances Sphk1 expression. By upregulating the Sphk1/S1P/S1PR1 axis and activating the NF-κB and STAT3 pathways in acute colitis, experimental data suggest that Sphk2 exerts anti-inflammatory effects (51). Sphk2 deficiency is associated with structural abnormalities and Th17 responses and does not exacerbate colonic inflammation caused by subchronic stress (52). It has been found that S1P produced by CD11b macrophages through Sphk2 can inhibit the Type 1 interferon gene stimulating factor (STING) signaling in alveolar macrophages, thereby alleviating acute lung injury (53). Interestingly, Sphk2 inhibitors have been found to have anti-inflammatory effects in autoimmune encephalomyelitis (54), and Opaganib (ABC294640) is a specific Sphk2 inhibitor that competitively binds to prevent S1P phosphorylation to its active form, thus effectively reducing intracellular S1P levels and restricting the inflammatory signaling pathway (55). Recently, in experimental studies on acute lung injury, Sphk2 was found to promote LPS-induced M1 macrophage polarization, oxidative stress, and NLRP3 inflammasome activation *in vitro* by regulating P53 acetylation. Sphk2 upregulation increases nuclear S1P levels. The Sphk2 inhibitor opaganib improved LPS-induced lung oxidative damage and inflammation (56). In addition, increased S1P levels in the blood of Sphk2^–/–^ mice were demonstrated to be due to the inability of Sphk2-dependent cells to degrade S1P in the blood, particularly in the liver, resulting in S1P accumulation in circulation (57, 58). Taken together, these results suggest that different manifestations of these phenomena are linked to different stimulation conditions and protein subcellular localization. Surprisingly, Sphk1^–/–^ and Sphk2^–/–^ mice displayed a trend toward reduced disease, improved survival of septic mice, and reduced release of pro-inflammatory cytokines, although Sphk1^–/–^ mice showed a 50% reduction in S1P in plasma. However, Sphk2^–/–^ mice depict a 2- to 3-fold increase in plasma S1P levels (59). IL-12 is a key immunomodulatory cytokine that promotes Th1 differentiation and cell-mediated immune responses, and Sphk2 is involved in IL-12 signaling by binding to the cytoplasmic region of IL-12β1 (60). Therefore, designing highly efficient and selective inhibitors using Sphk1/2 structure suggests a potential direction for treating autoimmune or inflammatory diseases.

## 4 Role of S1P in immune cell trafficking

### 4.1 S1P is involved in immune cell trafficking

**Figure 1:**
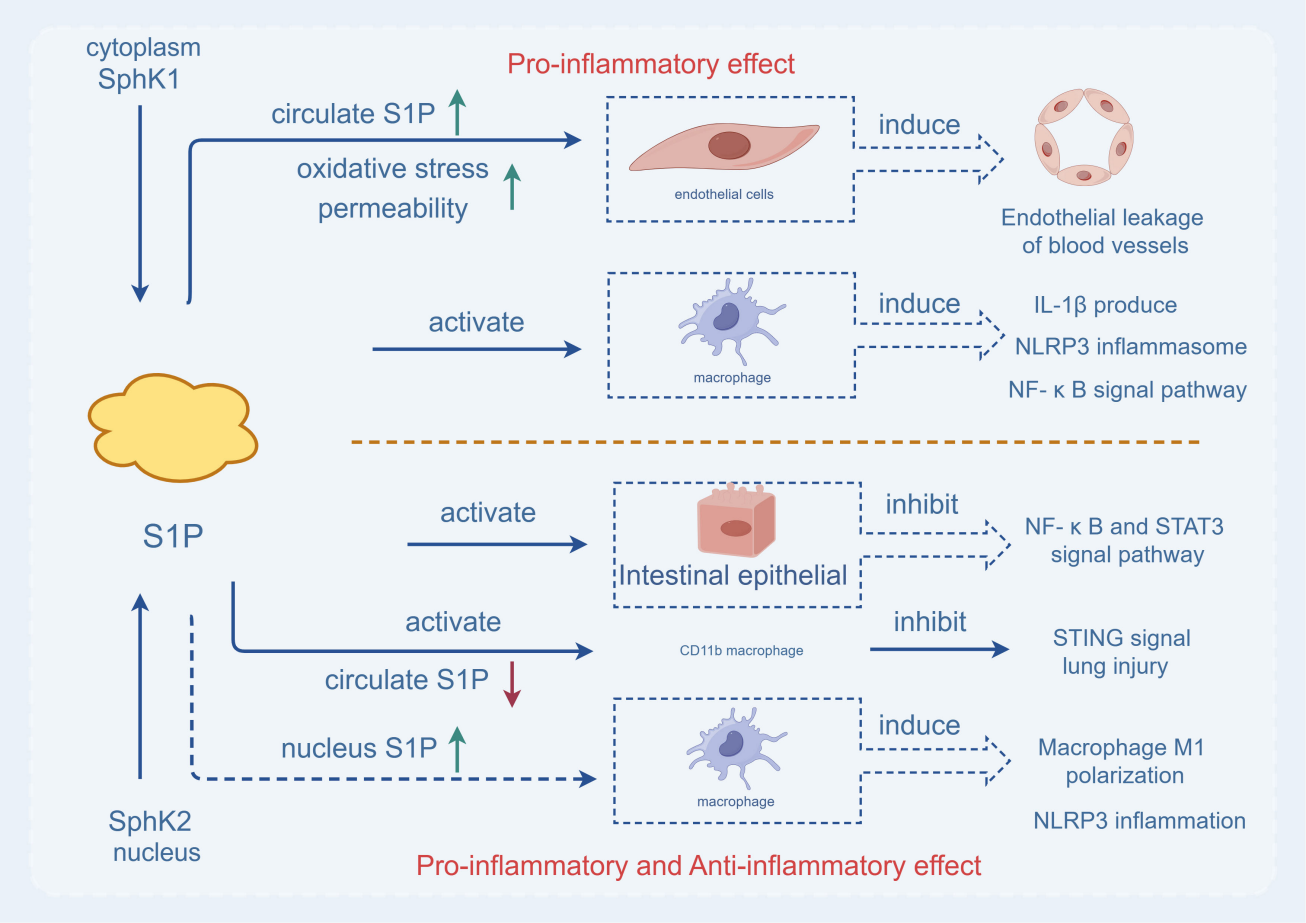
The two Sphks play different roles. Sphk1, which exists in the cytoplasm, is activated and produces S1P, which can directly act on macrophages, cause the production of interleukin 1-β, the release of NLRP3 inflammasome and the activation of pro-inflammatory signaling pathway (NF-κB pathway), aggravating inflammatory response. At the same time, under the action of SPHK1 in the cytoplasm, S1P increases the level of S1P in the blood circulation, oxidative stress leads to increased permeability, causes vascular endothelial leakage, and is characterized by pro-inflammatory effect. While Sphk2, which exists in the nucleus, is activated and produces S1P, which directly acts on intestinal epithelial cells, inhibits the activation of pro-inflammatory signaling pathway (NF-κB pathway), and reduces inflammatory response. SPHK2 can reduce the level of S1P in the blood circulation, act on CD11b macrophages, inhibit the STING signaling pathway to reduce acute lung injury. In addition, up-regulation of Sphk2 can increase the level of S1P in the nucleus, leading to M1 differentiation of macrophages and the increase of NLRP3 inflammasome. Therefore, SPHK2 has both pro-inflammatory and anti-inflammatory effects.

Initially, in 2002, researchers Mandala et al. demonstrated that S1P receptor inhibitor FTY720 could isolate T cells in lymph nodes (LN) instead of spleen cells, thus becoming a new immunosuppressive drug for transplant rejection through immunosuppression (61). Brinkmann et al. found that FTY720 induced lymphopenia by acting on S1PRs, excluding S1PR2, which was caused by a reversible redistribution of lymphocytes from the circulation to secondary lymphoid tissues, the mechanism of which is unknown (62). Besides, some studies have uncovered that Sphk1 and S1PR2 regulate the migration and degranulation of mast cells, and mast cells can secrete S1P, which is important in reducing inflammation and immediate allergic reactions (63). Subsequently, most studies on the effects of S1P on immunity mainly focused on the rapid and transient changes in lymphocyte migration and transport, as well as mast cell migration and chemical mediator secretion. Differential expression of S1PR subtypes was also explored, and it was confirmed that lymphocytes played an important role in S1P migration. Inhibiting lymphocyte recycling by activating S1PRs may provide new therapeutic prospects for immunosuppressive and inflammatory diseases (64–67). Subsequently, S1P and its receptors were found to be required for thymocyte migration from the thymus, trafficking of lymphocytes in secondary lymphoid organs, and migration of B cells to splenic follicles, demonstrating that S1PR1 is the major S1P receptor that regulates T cell trafficking and that S1PR1 promotes T cell trafficking at multiple stages of T cell development and response (68, 69). S1P regulates the migration of lymphocytes in LN by inhibiting the entry of chemokines into lymphocytes and the chemotactic stimulation of LN into efferent lymphocytes with higher concentrations of S1P (70). S1P binds to S1PR1 in perivascular cells and promotes the production of pro-inflammatory cytokines and chemokines in the injured state, leading to immune cell infiltration and fibrosis (71). S1P acts as both an intracellular messenger and an extracellular mediator in immunity, and the control of thymocyte migration, lymphocyte migration in secondary lymphoid organs, and chemotaxis of lymphocytes in non-immune tissues by S1P mainly depends on the cellular expression level of S1PR1 and the magnitude of the S1P concentration gradient (68, 72) (**Figure 1**, By Figdraw).

### 4.2 S1P gradient

S1P functions efficiently because its distribution is tightly controlled. The S1P gradient between the LN and lymph regulates the exit of immune cells from tissues. Many studies have demonstrated that the flow of T-cells between LN and lymph is in accordance with the S1P concentration gradient (from low to high concentration). T-cells travel from lymph to blood and finally to inflamed tissues, and S1P mainly follows the S1P gradient through S1PR1 (73). Lymphocytes exit the tissue and enter circulation following the S1P gradient through five G protein-coupled receptors. During the immune response, both S1P gradient and expression of S1P receptors are dynamically regulated (74). The interaction of S1P synthesis and degradation enzymes with S1P production creates a concentration gradient fundamental to S1P biology. High concentrations of S1P in the blood and lymph stabilize the vasculature while attracting immune cells into circulation, and understanding how S1P simultaneously directs multiple cell movements between tissues and circulation as well as within tissues is necessary for developing S1P-related specific targeted drugs (35). Comprehensive lipidomic analysis displayed that plasma S1P levels in Mfsd2b knockout mice decreased by 42%–54% compared with WT levels, indicating that Mfsd2b actively exports S1P and insufficient reduction of plasma S1P in knockout mice caused vascular leakage. Mfsd2b is essential for S1P export from erythrocytes and platelets (75). The redistribution of lymphocytes from the spleen to the LN and the loss of circulating lymphocytes in Spinster homologue 2 (Spns2)-deficient mice are consistent with plasma S1P-guided normal spleen export and lymphatic S1P-guided LN export blocked, and endothelial cells require Spns2 to supply lymphatic S1P and support lymphocyte circulation (76). In addition, using the S1P reporter, it was found that cells have a higher concentration of S1P in the medullary cord than in the T-cell zone and that the S1P transporter Spns2 on lymphatic endothelial cells produces this gradient (77).

**Figure 2:**
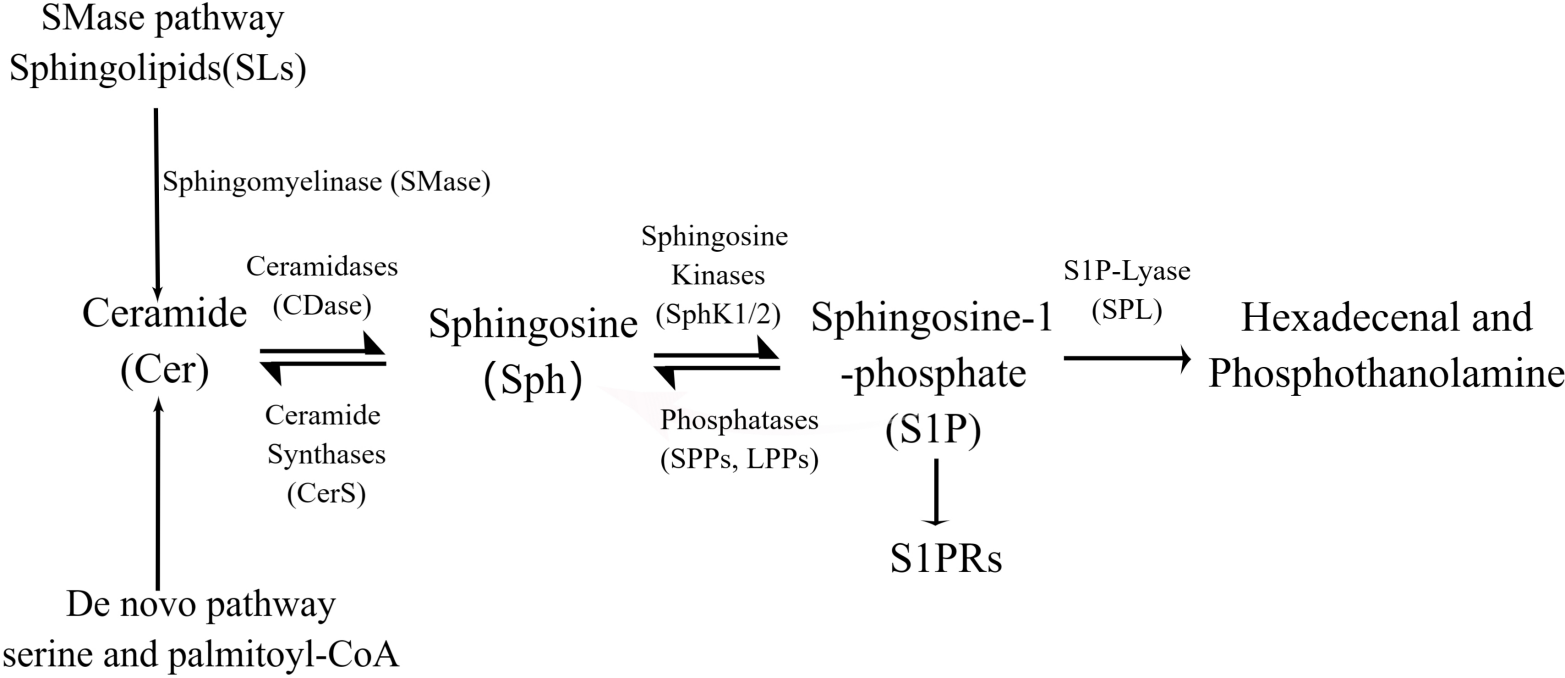
S1P is synthesized from serine and palmitoyl-CoA by a *de novo* pathway or from the ubiquitous membrane lipid sphingolipia (SMD). Sphingosine is produced by hydrolysis of ceramides, which can be recycled by acylation, a process known as the “rescue pathway” that can lead to ccramide regeneration. In the cell membrane and cytoplasm, sphinganine is converted to S1P by sphinganine kinases (SphKs). Finally, SIP is cleaved by SIP cleaver (SPL) or dephosphorylated to hexadecaneal and phosphothanolamine.

The complex metabolism of S1P is important for the formation of the S1P concentration gradient, and the metabolic map of active sphingolipids (ceramide, sphingosine, and S1P) shows the relationship between upstream and downstream pathways of S1P and each sphingolipids (**Figure 2**, By Figdraw). Ceramidase and SPHKs are key enzymes in S1P synthesis, and it has been shown that these key enzymes are the basis for the modulation of S1P proinflammatory activity. Injection of acid ceramidase alleviates liver ischemia-reperfusion (IR) injury. And increased after pretreatment with acid ceramidase (78). STING is a major regulator of innate immunity and is involved in a variety of inflammatory diseases. After activating STING, blocking ceramidase and SPHK I/II can significantly reduce IL-6 (79). Moreover, inhibition of ceramidase and SPHK I/II by S1P significantly reduced TLR9-induced TNF-α release (80). SPHKs inhibitor could completely eliminate the release of TNF-α and IL-6 by S1P-stimulated Peripheral blood mononuclear cell (PBMC) derived from lung cancer (81).Yugesh Kharel et al. revealed that mice lacking Sphk2 or S1P degrading enzymes were used to reveal an S1P gradient mechanism whereby S1P is dephosphorylated on the surface of hepatocytes, and the resulting Sph is phosphorylated and chelated by Sphks and then degraded by intracellular SPL (58). S1P concentration is maintained in a gradient by the activity of S1P degrading enzymes, which is essential for lymphocyte exit (82). Analysis of ceramide synthase 2 (Cers2)-deficient mice revealed that Cers2 restricts S1P levels in the thymus and blood to maintain a functional S1P gradient that mediates thymocyte migration into circulation (83). Moreover, SPL deletion in dendritic cells (DC) disrupted the S1P gradient, proving that SPL in DC regulates thymic exit (84). These experiments illustrate the complexity of S1P metabolism and the sensitivity of the thymic outlet to the disruption of the S1P gradient, which is also regulated by enzymes involved in the synthesis, export, and degradation of S1P. However, there are few studies on the distribution of S1P in diseases or how changes in S1P levels affect immune response. S1P is usually derived from red blood cells and endothelial cells (85). Recent studies have shown that hematopoietic cells and inflammatory monocytes (iMo) are important sources of S1P in LN during the immune response, and S1P levels increase during the immune response. iMo requires the early activation marker CD69 to provide this S1P, which acts as a “stand your feet” signal to maintain immune cells at the site of inflammation by regulating S1P receptors and gradients, prolonging the residence time of T cells in LN (86). The S1P gradient between tissues and the circulatory system plays a key role in regulating the trafficking of immune cells such as autoreactive B and T lymphocytes. S1P receptor modulators may be a safe and effective alternative mechanism to reduce inflammation in immune-mediated diseases by reducing the exit of lymphocytes from the lymph nodes to the blood (74).

## 5 immune role of S1P in inflammation

### 5.1 innate immunity

**Figure 3:**
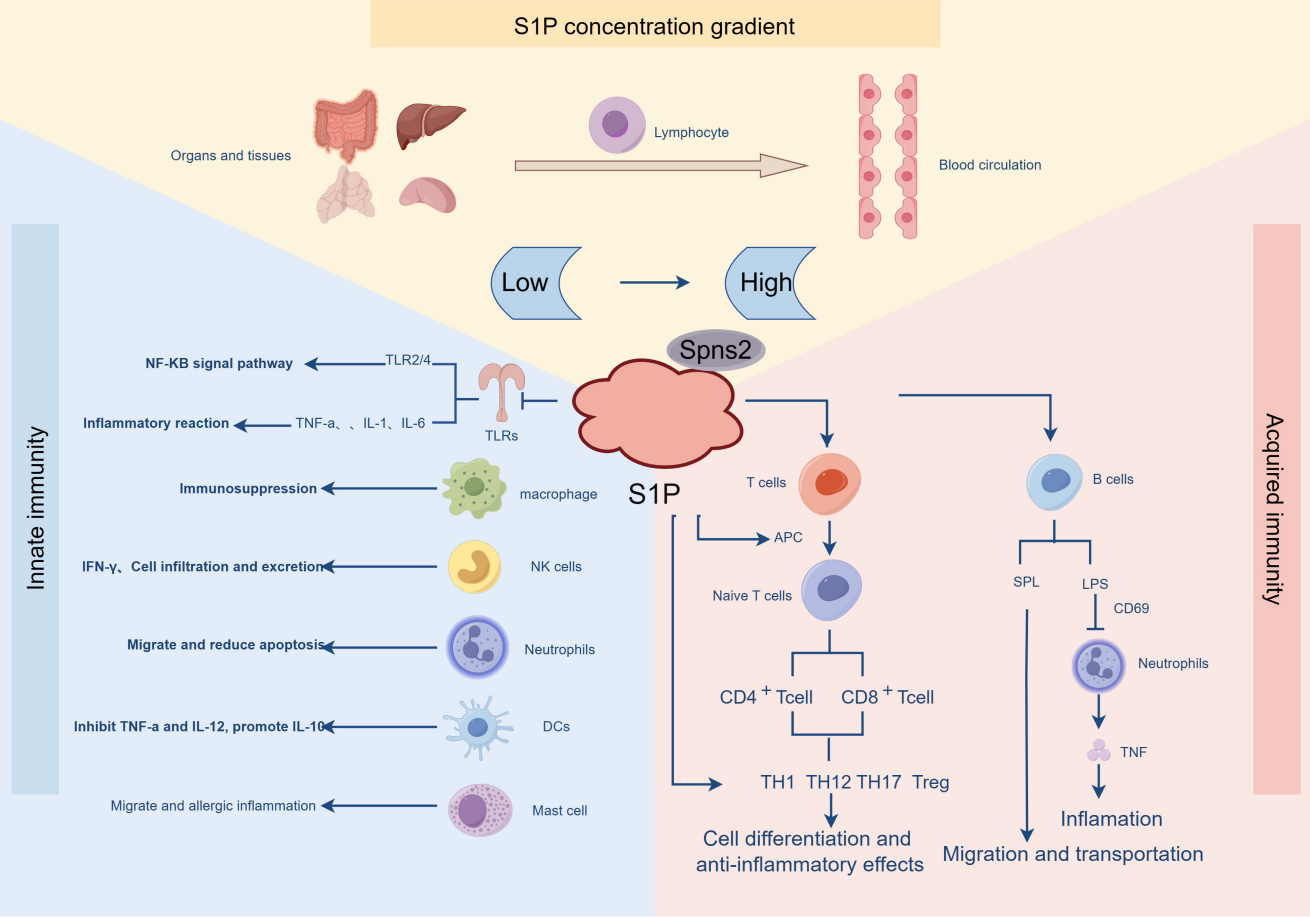
The distribution of S1P is strictly controlled and involved in innate and adaptive immunity. S1P is distributed in low concentration in organ tissues, and in the blood circulation, S1P presents the characteristics of high-level distribution. In the innate immune response, S1P plays a role through the receptors on various immune cells, such as in TLRS, activating the NF-KB signaling pathway, promoting the release of a variety of pro-inflammatory factors, inhibiting immune responses in macrophages, and reducing migration and apoptosis in neutrophils. In the adaptive immune response, under the action of APC, T cells are differentiated and produce anti-inflammatory effects. In addition, S1P inhibits LPS-induced B cell inflammatory responses and promotes B cell migration and transport.

S1P can regulate the innate immune system to fight pathogens, and for many S1P-regulated immune cells, the diversity of their cellular functions can be explained by the expression repertoire of S1PRs in various immune cells (**Figure 3**, By Figdraw). The innate immune system is the first line of host defense against foreign stimuli. The innate immune cells (macrophages, natural killer cells, neutrophils, DC cells, etc.) are activated by danger signals. Pathogen-associated pattern recognition receptors (PRRS) activate ligands and stimulate the secretion of effector molecules (cytokines and antimicrobial peptides), thereby inducing inflammatory responses and eliminating pathogens (87). Spns2 is a major transporter of S1P, and Fang et al. demonstrated in a rat sepsis model that Spns2 regulates inflammation through the lactic-mitochondrial reactive oxygen species (ROS) axis and is a key mediator of macrophage metabolic reprogramming during sepsis (88). Moreover, attenuated Spns2/S1P signaling impairs the ability of macrophages to maintain antimicrobial responses, leading to significant innate immune suppression in the later stages of infection. Consequently, enhancing Spns2/S1P signaling helps balance the inflammatory imbalance and immune response during sepsis (89). Studies on glial cells have disclosed that excessive S1P can lead to neuroinflammation, NF-κB activation, and macrophage infiltration into the central nervous system, and S1P inhibition has become an effective target in multiple sclerosis (90). Immune cell-driven inflammation is a key determinant of nonalcoholic steatohepatitis (NASH) progression. Mauer et al. demonstrated that inhibiting pro-inflammatory monocyte chemotaxis using the S1P antagonist FTY720 ameliorated NASH-induced liver injury, inflammation, and fibrosis (91). Recently, using etrasimod, an antagonist of S1PR1, S1PR4, and S1PR5, the team found that etrasimod reduced the accumulation of activated macrophages in the liver, as well as the infiltration of inflammatory cells (T, B, and NK cells). Ultimately, it reduces liver injury and inflammation (92). Natural killer (NK) cells are an important component of the innate immune system and can quickly attack target cells. Studies have found that the expression of SPNS2 and S1PR5 in NK cells is required to achieve homeostasis and the rapid production of interferon-γ (77). NK cells are located in the periphery of LN, and S1PR5 expression is upregulated during NK cell maturation, while the number of NK cells in BM and LN of S1PR5-deficient mice is doubled, and the effect of S1PR5 on NK cells (but not T and B cells) efflux from BM and LN is specific (93). In addition, many neutrophil infiltration and neutrophil extracellular trap (NET) formation have been confirmed to be linked to chronic inflammation. *In vitro* experiments have demonstrated that S1P significantly activates neutrophils, prolongs the half-life of neutrophils, and delays neutrophil apoptosis (94). In mice and patients with chronic hepatitis, Sphk1 or S1P levels are positively correlated with the expression of neutrophil markers, and S1P significantly promotes the migration and cytoskeleton remodeling of bone marrow neutrophils through S1PR1 or S1PR2 and plays a key role in neutrophil recruitment (95).

S1P-activated neutrophils (A-Neu) suppressed pro-inflammatory cytokines (C-C chemokine motif ligand 4, tumor necrosis factor, and nitric oxide synthase 2) in the liver by suppressing the pro-inflammatory macrophage response. Tumor necrosis factor and nitric oxide synthase expression reduced liver inflammation, while the absence of activated neutrophils (N-Neu) did not (96). During development, natural killer (NK) cells leave the BM and reach the bloodstream. CXCR4 retains NK cells in the BM, while sphingosine-1-phosphate receptor 5 (S1P5) promotes their exit from the organ. During NK cell differentiation, CXCR4 expression decreases while S1P5 expression increases, thus facilitating the expulsion of mature NK cells from the BM (97). Previous studies have depicted that S1P may regulate the transport of DC. In mature DC, S1P inhibits the secretion of tumor necrosis factor-α and IL-12 but enhances the secretion of IL-10, which is ultimately conducive to Th2 lymphocyte-induced immunity (98). Recent studies have disclosed that S1PR4 is required for the activation of plasmacytoid dendritic cells (pDC) and that S1PR4 agonists block the activation of human plasmacytoid dendritic cells (pDC), thereby reducing TLR-induced IFN-α secretion, which may be useful for treating pathogenic IFN-α diseases (99, 100). FTY720 application also showed that S1P is involved in the activation of pDC and the expression of type 1 IFN to change pDC function (101). A series of intense struggles ended with dead immune cells and tissue cells, and some immune cells, such as neutrophils, began programmed death and apoptosis in the immune context, heralding the end of the disease struggle and the beginning of regeneration.

The innate immune response can also be further shaped by other receptor systems, such as cytokine receptors, some of which are closely linked to S1P-involved inflammatory responses, including Toll-like receptor (TLR) signaling, TNF receptor (TNFR)-dependent NF-κB activation, protease-activated receptor 1 (PAR1) signaling, FcϵRI in mast cells, and other receptors. Activation of TLRs causes the release of pro-inflammatory cytokines, such as tumor necrosis factor (TNF-α) interleukins IL-1 and IL-6, resulting in a strong inflammatory response, leading to sepsis and septic shock (102). It was initially found that S1P selectively attenuates TLR2 signaling, such as transcriptional activity driven by NF-κB signaling, and negative crosstalk between S1PRs and TLR2 signaling may be associated with the atherosclerosis protective effect of S1P (103). The SPHKs/S1P/S1PRs axis regulates many cellular processes, including proliferation, invasion, metastasis, and angiogenesis. Sphk1 expression is upregulated in peripheral blood monocytes of patients with sepsis (45), and studies have found that Sphk1 promotes inflammation and proliferation of glioblastoma through the NF-κB/IL-6/STAT3 signaling pathway (104). Recent studies have demonstrated that relieving ER stress by inhibiting Sphk1/S1P and TLR4/NF-κB signaling and reducing pro-inflammatory cytokine levels can significantly reduce lung inflammation caused by LPS infection (105). These results suggest that Sphk1 plays a key role in TLR signaling and identifies Sphk1 as a key target for treating the inflammatory cascade (45, 106). Coagulation triggered by PAR1 signaling cytokines during inflammation is a hallmark of systemic inflammatory response in bacterial sepsis. The pro-inflammatory signal of PAR1 destroys the integrity of the endothelial barrier, while S1P has been shown to have an effective barrier protective effect, and activated protein C protects the endothelial barrier through PAR1-dependent S1PR1 cross-activation (107). S1P regulates PAR1-mediated human platelet activation in a concentration-dependent biphasic manner, with the S1PR1 receptor having an activating effect, while the activation of S1PR4 and S1PR5 receptor has an inhibitory effect (108).

Dendritic cells promote systemic coagulation, and studies have found that the loss of PAR1-S1PR3 signaling in DC sequesters DC and inflammation into the draining LN and weakens IL-1β dissemination to the lungs, participating in systemic inflammation and coagulation processes in the innate immune response (109). Mast cells cross-link Sphk to produce S1P through the high-affinity receptor IgE Fc receptor (FcϵRI). This process plays an important role in mast cell function and may be involved in the movement of mast cells to inflammatory sites (110). FcϵRI signaling is necessary for allergic inflammation because FcϵRI activates SPHKs and increases S1P levels (111). Once S1P is secreted, it can bind to S1P receptors (S1PRs). Moreover, studies have found that FcϵRI/S1P signaling can mediate allergic reactions through the interaction between mast cells (MC) and macrophages (111).

### 5.2 Specific immunity

Specific immunity is an immune function acquired by stimulating internal and external environmental factors, which only plays a role against a specific pathogen and requires the participation of highly differentiated tissues and cells. The maintenance of tissue and cell homeostasis and the establishment of adaptive immunity after the inflammatory process are key events (112). Signaling through S1P-S1PRs is involved in various aspects of inflammatory cell functions. Unique features of S1PRs are expressed by T and B lymphocytes and endothelial cells. The first is the sensing phase, in which a central feature of the immune response is the precise spatiotemporal distribution of T cells and antigen-presenting cells (APCs) in specific microenvironments within secondary lymphoid organs (SLOs). Initially, researchers observed that S1PR agonists affect the entry and exit of T-cells into and out of LN, and direct contact between APC and T cells leads to naive T-cell initiation under the action of chemokines. Molecular studies on how T cells and APC contact and migrate within the SLO are still being explored (113). Both S1PR1-S1PR5 are involved in regulating APC function and metabolism (114, 115). Although the circulation of APC is mainly not regulated by the S1P system, FTY720 can affect the migration of APC to LN and tissues by regulating inflammatory chemokines (113, 116, 117). Subsequently, increasing evidence has highlighted that S1P is closely linked to the metabolism and immunity of APC, and FTY720 acts as an immunomodulatory agent to reduce the pro-inflammatory ability of APC, a process that is a key factor in DC-dependent T-cell activation and programming (118). The second reaction stage is antigen-binding B-cell antigen receptor (BCR) or MHC-mediated antigen presentation to activate the T-cell receptor (TCR), which activates multiple signaling cascades in B and T cells, respectively (119). Most CD4 ^+^ T cells develop from the thymus into conventional T cells or naive T cells. Upon antigen stimulation, naive T cells differentiate into CD4 helper cells, CD8 cytotoxic effector cells, and memory cells. Th17 and iTreg cells, which mediate direct killing and various immune regulatory functions and exert specific effector functions (120, 121). CD4 helper T cells are central regulators of adaptive immune responses, which can promote immune activation and induce tolerance and are essential for immune defense (119). The differentiation of TH1 cells and anti-inflammatory Foxp3 (+) Treg cells is mutually regulated by S1PR1, which inhibits the production of extrathymic and natural Treg cells while driving TH1 development and disrupting immune homeostasis (122). S1PR1 blocks the differentiation of thymic Treg precursors and induces selective activation of the Akt-mTOR kinase pathway to impede Treg cell development and immune tolerance (123). S1PR1 through S1P analog experiments showed that three analogs directly inhibited Th1 cell differentiation *in vitro* and increased Treg differentiation of naive CD17 ^+^ T cells. In addition, all three S1P analogs inhibited IL-1-mediated activation of STAT3, NF-kB, and AKT (124). One study demonstrated that S1PR1-mediated TH17 polarization depends on sustained S1PR1 signaling in bone marrow cells, exacerbating the severity of neuroinflammation independent of intrinsic T-cell effects (125). In the effector stage, effector T cells, antibodies, and lymphokines exert immune effects. Lymphatic endothelial cells (LECs) present peripheral tissue antigens to induce T cell tolerance, and LECs are the major source of S1P, which promotes naive T cell survival and effector T cell ejection from LN (126). S1P can selectively enhance the migration of human and mouse CD4 ^+^ T cells in LECs, and S1PRs play different roles in the migration process. For example, S1PR1 and S1PR4 differentially regulate T-cell motility and vascular cell adhesion molecule-1 (VCAM-1) binding. S1PR2 regulates LEC layer structure, permeability, and expression of VE-cadherin, occludin, and zonulin-1 through the ERK pathway and promotes transcellular migration of T cells (127). B-cell migration within LNs is important for adaptive immune responses, and the S1P gradient has been found to be the driver of B-cell trafficking, which mediates B-cell egress in LN through SPL expression (128). Moreover, in isolated B cells, S1PR1 agonists inhibited neutrophil accumulation in mice with endotoxin-induced hypersensitivity pneumonia and reduced TNF production by B cells and their ability to trigger T cell cooperation *in vitro (* 129).

## 6 S1P receptor modulators in inflammatory diseases

### 6.1 Genomic studies

Proteomics analysis can effectively assess the changes in the proteome that occur during sepsis. Multiple genomic studies have disclosed the role of lipids in pathogen toxin clearance and regulation of the inflammatory response. Furthermore, S1P apolipoproteins decrease, and lipid changes are associated with sepsis and systemic inflammation (130–132). In genome-wide association studies, the most significant effect on sphingolipid levels was the reduction in circulating S1P levels. LPS treatment reduced neutrophil survival in a time-dependent manner, whereas S1P treatment inhibited the effects of LPS and enhanced neutrophil survival in mice. All suggest that circulating S1P can regulate neutrophil survival and recruitment after LPS-induced airway inflammation (133). Multi-omics analysis has characterized the ceramide/sphingomyelin pathway as a therapeutic target for Alzheimer’s disease, and mouse experiments have depicted that long-term exposure to fingolimod alleviates synaptic plasticity and cognitive impairment in mice, and modulators of S1P metabolism have become possible candidates for Alzheimer’s disease treatment (134). In human umbilical vein endothelial cells, viral genome analysis revealed that HSV-1 viral genome replication depends on sphingosine kinase activity and S1PRs (S1PR1,3-5) signaling, which involves the activation of PI3K and Rac-1. Targeting S1P-related signaling may be a successful strategy for establishing new anti-HSV-1 therapies (135). Severe trauma can trigger systemic inflammatory response, leading to infection, sepsis, or multi-organ failure. Based on the genome-wide screening of ten representative patients with severe trauma or known immune response mechanisms, transcriptome researchers have found that SPHK1 expression on the first day of admission indicates mortality and has become a marker (106). Furthermore, the critical role of S1P/SPHK2 signaling in promoting Pseudomonas aeruginosa pneumonia was demonstrated in SPHK2^–/–^ mice and differential gene expression analysis, with SPHK2 promoting inflammation and inhibiting other anti-inflammatory and host defense genes (136).

### 6.2 Targeted therapeutic strategies of S1P receptor modulators

Currently, therapeutic molecules targeting S1PRs can be divided into two categories: lipid S1P mimetics, such as non-selective FTY720 (fingolimod), or non-lipid molecules, such as the clinical drugs siponimod (S1PR1, 5), ozanimod, and CBP-307 (S1PR1, 4, and 5) (137). Fingolimod, a first-generation S1PR modulator, has been approved by the FDA for treating multiple sclerosis, but the drug has poor target selectivity and widely acts on S1PR1-5; accordingly, the side effects are relatively large, mainly due to the drug binding to S1P receptors rather than S1PR1 (138–140). The second-generation S1PRs modulators, ozanimod, estrasimod, and CBP-307, have been optimized for receptor isoform selectivity and have very low affinity for S1PR3. Therefore, side effects such as pro-inflammatory and pulmonary fibrosis are avoided to a certain extent (26, 141).

FTY720 has been approved and is currently used for MS treatment. Modulating S1PRs by FTY720 in mouse and human astrocytes inhibits pathogenic astrocyte activation and chronic progressive central nervous system inflammation. Moreover, it is effective for secondary progressive MS (SPMS) (124, 142). FTY720 regulates gene expression in inflammation and amyloid-β metabolism and improves exploratory and anxiety-like behaviors in obese mice. It has a positive effect on reducing inflammation-driven neurodegeneration (143). Consequently, therapies focusing on the S1P pathway can also be used to treat autoimmune diseases rather than MS. Siponimod and ozanimod have also been employed in treating recurrent MS or ulcerative colitis recently, and these drugs slow down the inflammatory progression of the disease through immunomodulatory effects (144, 145). Siponimod regulates microglial cytokine gene expression, significantly reduces LPS-induced TNF-α and IL-1β, and is involved in regulating the immunological characteristics of microglia triggered by pro-inflammatory stimulation (146). Ozanimod can reduce the secondary inflammatory response induced by cerebral hemorrhage by regulating the AIM2 inflammasome mediated by the SIRT2/NF-κB/AIM3 pathway, indicating that ozanimod may become a targeted therapy to improve the prognosis of cerebral hemorrhage (147). S1PR modulators have become a hot topic in treating inflammatory bowel disease. S1PR modulators can treat IBD by inhibiting S1P-S1PR1 signal transduction, thereby inhibiting lymphocyte infiltration into the inflamed intestinal lamina propria. CBP-307 is a highly potent and selective S1PR1 modulator currently being evaluated in a global phase 2 clinical study of moderate-to-severe ulcerative colitis and Crohn’s disease (148). In the latest phase 3 drug trial, etrasimod (S1PR1, 4, and 5) was used as an S1P receptor modulator for treating immune-mediated diseases, including ulcerative colitis, confirming the efficacy of etrasimod as induction and maintenance therapy in adult patients with moderately to severely active ulcerative colitis (149).

In sepsis, miR-145 improves the contractile response of vascular endothelial cells mainly by activating the phosphorylation of Sphk2/S1PR1/myosin light chain 20 pathway. However, miR-132 can effectively improve the barrier function of vascular endothelial cells by activating Sphk2/S1PR2/ZO-1 and the vascular endothelium-cadherin pathway (150). In gynecological diseases, including endometriosis, adenomyosis, and uterine fibroids, which are characterized by inflammation and fibrosis, the Sphks-S1P-S1PRs pathway plays a role in increasing endometriotic cell growth. S1P promotes fibrosis in cells such as macrophages, fibroblasts, and skeletal muscle precursors, mainly by binding to pro-inflammatory cytokines such as TNF-α. In turn, it stimulates the synthesis of interleukin-1β and TGF-β in cells (151). The disruption of S1P pathway is the basis of systemic chronic metabolic inflammatory diseases, including diabetes and gastrointestinal cancer, which provides sufficient evidence for using S1P pathway modulators in treating pathological inflammation (152). Considering these findings, the modulation of S1P signaling may represent an innovative and promising therapeutic target for inflammatory diseases.

## 7 The future and outlook

Bioactive sphingolipid metabolite S1P is one of the key sphingolipids involved in innate and adaptive immune responses and is closely related to the activation, differentiation, and trafficking of immune cells. S1P and S1PRs have been identified as key players in the maintenance of immune homeostasis and pathophysiological processes in inflammatory diseases. As the basis of most diseases, inflammation increases the risk of many chronic disease states. Since the broad expressions of S1P and S1PRs are universally expressed in different tissues, S1P signaling can be pro-inflammatory or anti-inflammatory, depending on the context and tissue of origin. According to the current research results, most researchers point out that S1P is involved in the body’s immune defense as a protective factor. In the future research, the exploration of the biological function of S1P is still a hot topic, and the application and transformation of S1P is also the ultimate research goal. At present, it is generally believed that S1P affects the inflammatory immune response by binding to S1PR1 on lymphocytes and regulating the migration of T and B cells from peripheral lymphoid tissues. Accumulating experimental evidence suggests that S1P is a key molecule controlling different physiological processes and is essential for normal and pathological conditions, including inflammation, autoimmune diseases, and fibrosis such as fibrosis of organs, MS, cardiovascular diseases, and cancer. So far, clinical trials of drugs for autoimmune diseases have shown promising results, and the preliminary related research of S1PRs as prevention of chronic inflammatory diseases is very extensive, which is of great guiding significance for developing inflammatory immune modulators, and S1PRs will have broad prospects in the future market.

## Author contributions

GS: Conceptualization, Data curation, Writing – original draft, Writing – review & editing. BW: Investigation, Software, Writing – review & editing. XW: Data curation, Formal Analysis, Writing – review & editing. JC: Conceptualization, Data curation, Writing – review & editing. JY: Methodology, Supervision, Writing – review & editing. CW: Conceptualization, Data curation, Writing – review & editing. HZ: Data curation, Supervision, Writing – review & editing. LX: Data curation, Methodology, Supervision, Writing – review & editing.
